# UV-triggered p21 degradation facilitates damaged-DNA replication and preserves genomic stability

**DOI:** 10.1093/nar/gkt475

**Published:** 2013-05-30

**Authors:** Sabrina F. Mansilla, Gastón Soria, María Belén Vallerga, Martín Habif, Wilner Martínez-López, Carol Prives, Vanesa Gottifredi

**Affiliations:** ^1^Cell Cycle and Genomic Stability Laboratory, Fundación Instituto Leloir-CONICET, Buenos Aires C1405BWE, Argentina, ^2^Instituto Clemente Estable, Montevideo 11600, Uruguay and ^3^Department of Biological Sciences, Columbia University, New York, NY 10027, USA

## Abstract

Although many genotoxic treatments upregulate the cyclin kinase inhibitor p21, agents such as UV irradiation trigger p21 degradation. This suggests that p21 blocks a process relevant for the cellular response to UV. Here, we show that forced p21 stabilization after UV strongly impairs damaged-DNA replication, which is associated with permanent deficiencies in the recruitment of DNA polymerases from the Y family involved in translesion DNA synthesis), with the accumulation of DNA damage markers and increased genomic instability. Remarkably, such noxious effects disappear when disrupting the proliferating cell nuclear antigen (PCNA) interacting motif of stable p21, thus suggesting that the release of PCNA from p21 interaction is sufficient to allow the recruitment to PCNA of partners (such as Y polymerases) relevant for the UV response. Expression of degradable p21 only transiently delays early replication events and Y polymerase recruitment after UV irradiation. These temporary defects disappear in a manner that correlates with p21 degradation with no detectable consequences on later replication events or genomic stability. Together, our findings suggest that the biological role of UV-triggered p21 degradation is to prevent replication defects by facilitating the tolerance of UV-induced DNA lesions.

## INTRODUCTION

The levels of the cyclin kinases inhibitor, p21, increases after treatment with various genotoxic agents, including γ irradiation, daunorubicin and others. Increased p21 expression inhibits the activation of cyclin-dependent kinases (CDKs), blocks cell proliferation and promotes cell survival and genomic stability by providing a temporal window for DNA repair [reviewed in (1)].

In contrast, p21 is downregulated by other DNA-damaging agents, including UV irradiation [reviewed in (1,2)]. UV irradiation selectively enhances the degradation of the p21 pool bound to proliferating cell nuclear antigen (PCNA) by activation of CRL4Cdt2 ubiquitin ligase complex (3,4). We have recently proposed that PCNA-coupled p21 proteolysis could facilitate the interaction of PCNA with a different set of partners (1). The PCNA-interacting domain of p21 (PIP box) is strong and is able to displace replicative pols from PCNA *in vitro* (5–8). As a direct implication, it was assumed that p21 degradation after UV would facilitate every DNA synthesis process associated to PCNA. Although some reports suggest that this is the case [reviewed in (2)], we have observed that persistent PCNA interaction with a p21 mutant that resists UV-induced degradation does not affect replicative or repair-associated unscheduled DNA synthesis (9). In contrast, p21 stabilization impairs the interaction of PCNA with the specialized polymerase η (pol η), thus suggesting that p21 displaces pol η from PCNA complexes more efficiently than other replication or repair factors (1,9).

Pol η is a member of the Y family pols, which participates in translesion DNA synthesis (TLS), a replication auxiliary process that uses damaged DNA as a template. Interestingly, TLS is facilitated by PCNA ubiquitination at sites of DNA damage (10), and we have shown that p21 stabilization impairs PCNA ubiquitination after UV irradiation (9). In apparent contradiction, Avkin and colleagues proposed that p21 might facilitate TLS, as UV-induced PCNA ubiquitination is impaired after p21 knockdown in U2OS cells (11). They showed that p21 negatively regulates the efficiency and increases the accuracy of gap-filling TLS events, which are uncoupled from replication forks (11) and conclude that TLS is facilitated by a p21-dependent increase in PCNA ubiquitination, which promotes the selection of the less mutagenic Y polymerase (pol η in the case of UV irradiation) (11,12). Hence, although a connection between p21, PCNA and pol η was previously established, it remains unclear whether p21 facilitates or represses TLS events (coupled or uncoupled with replication forks).

Herein, we explore the relationship between p21, PCNA and Y polymerases function during the replication of UV-damaged DNA. We took advantage of a stable p21 mutant that does not alter cell cycle progression because of a disrupted CDK binding site (from here on referred as sp21^ΔC^). In UV-irradiated sp21^ΔC^-expressing cells, persistent p21/PCNA interaction caused the accumulation of molecular markers of DNA damage such as the phosphorylation of histone H2AX (γH2AX), 53BP1 focal organization and increased S phase-associated genomic instability, as revealed by micronuclei (MN) formation. Persistent p21/PCNA interaction also abolished the focal organization of specialized Y polymerases, impaired damaged DNA replication and subsequent S phase progression. In contrast, degradable p21 expression only transiently delayed replication events and Y polymersases focal organization in a manner that correlated with its degradation. This suggests that the PCNA bound to p21 at replication forks might serve to control the timing of TLS onset. Remarkably, degradable p21 (even when highly overexpressed) neither affected S phase progression at later time points after UV irradiation nor did it alter markers of stress or MN formation. Thus, our data indicate that the timely removal of p21 from replication forks is a crucial event during the response to damaged DNA accumulation after UV irradiation.

## MATERIALS AND METHODS

### Cell culture, expression vectors and UV irradiation

U2OS, Hela cells (ATCC) and HCT116 p21+/+ and HCT116 p21−/− (obtained from B. Vogelstein-Johns Hopkins University, Baltimore) were grown in Dulbecco’s modified Eagle’s medium (Invitrogen) with 10% fetal calf serum. Transfections were performed using Lipofectamine 2000 (Invitrogen) and Jet Prime (VWR).

In this work, we used or modified the following expression vectors: p21 and sp21^ΔC^ (CS2-p21 and CS2MT-p21(CDK-) previously generated by us (9,13); GFP-Pol η and GFP-pol κ were gifts from Dr A. Lehmann (14); GFP-pol ι was provided by Dr R. Woodgate and GFP-Rev1 was donated by Dr Friedberg. To generate CDK- and PCNA-binding mutations in p21 expression vectors, quick-change site-directed mutagenesis (Stratagene) were used. Primers used are described in the Supplementary Material section.

Ultraviolet light C (UVC) irradiation was performed using a CL-1000 ultraviolet cross-linker equipped with 254 nm tubes (UVP) orXX-15S UV bench lamp from UVP. For full cell irradiation, doses from 3 to 50 J/m^2^ were delivered after removal of the culture media. For local irradiation, polycarbonate filters containing multiple 5 μm pores (Millipore # TMTP01300) were positioned in direct contact with cells and subjected to 120 J/m^2^ [equivalent to a much lower dose as reported in (15)].

### Immunostaining and microscopy

For the quantification of specialized Y polymerases, 53BP1 foci and the determination of γH2AX intensity, cells were fixed in 2% paraformaldehyde/sucrose for 20 min followed by 15 min incubation with 0.1% Triton X-100 in phosphate buffered saline (PBS). For the detection of replicative DNA synthesis with Bromodeoxyuridine (BrdU) incorporation (10 uM-SIGMA) by immunofluorescence, GFP-PCNA was used as transfection marker, as it resists the denaturation procedure required to expose the BrdU epitope (HCl 1.5N for 5 min). Similar results were obtained when using EdU (10 uM-Invitrogen) and f-GFP as a transfection marker. EdU-treated cells were fixed in PFA (2%) and subjected to EdU detection following manufacturer’s instructions (Click-iT®EdU kit – C10338). Blocking was performed overnight in PBS 2% donkey serum (SIGMA). Coverslips were incubated for 1 h in primary antibodies: α p21 AB1 (Oncogene Research Products), α p21 C19 (Santa Cruz), α BrdU (Amersham), α γH2AX (Upstate), α 53BP1 (Santa Cruz), α CPDs (MBL international corporation). Secondary anti-mouse/rabbit-conjugated Cy2/Cy3 antibodies were from Jackson ImmunoResearch and anti-rabbit alexa 488 from Invitrogen. GFP-tagged specialized Y polymerases were detected by GFP auto-fluorescence. Nuclei were stained with DAPI (SIGMA). Images were obtained with a Zeiss Axioplan confocal microscope. Quantification procedures for γH2AX are described in the Supplementary Methods section.

### Cell cycle analysis

Cells were fixed with ice-cold ethanol and resuspended in phosphate-buffered saline containing RNase I (100 mg/ml, Sigma) and propidium iodide (50 mg/ml, Sigma). Samples were subjected to fluorescence activated cell sorting (FACS, Calibur, Becton Dickinson), and data were analyzed using the Summit 4.3 software (DAKO Cytomation), and cell cycle profiles were drawn using the FlowJo software (Tree Star Inc.). The profiles shown were obtained by gating the f-GFP positive cells by dual channel FACS analysis.

### MN assay

Transfected cells were replated at low density. The day after UV irradiation cytocalasine B (4.5 ug/ml-Sigma) was added to the media and 40 h later cells were washed 1 min with hypotonic buffer (KCl 0.0075 M) diluted 1/10 from stock solution in PBS 1X, twice with PBS 1X and fixed with PFA/sucrose 2% for 20 min. GFP-PCNA or farnesylated f-GFP were used to identify transfected cells obtaining similar results. DAPI was used to stain the nuclei. When analyzing MN formation, replicating cells were labeled with 10 µM EdU for 1 h immediately after UV irradiation. After fixation, EdU detection was performed as suggested by manufactures [Click-iT®EdU kit (C10338 from Invitrogen)] before DAPI stainning.

### Preparation and immunolabelling of DNA fibers

DNA fibers were analyzed using a protocol previously used by us (16). Briefly, exponentially growing cells were pulse labeled with CldU (20 µM) for 20 min, washed twice, irradiated with 20 J/m^2^ UVC and incubated with IdU (200 µM) for additional 20 min. Cells were trypsinized and lysed with 6 µl of 0.5% SDS, 200 mM Tris–HCL (pH 7.4) and 50 mM EDTA buffer onto clean glass slides, which were tilted, allowing DNA to unwind. Samples were fixed in 3:1 methanol/acetic acid and denatured with HCL (2.5 N) for 1 h, blocked in PBS 5% Bovine serum albumin (BSA) for 15 min and incubated with the mouse anti-BrdU (Becton Dickinson) to detect IdU, donkey anti-mouse Cy3-conjugated secondary antibody (Jackson Immuno Research), rat anti-BrdU (Accurate Chemicals) to detect CldU and donkey anti-rat Alexa 488 secondary antibody (Invitrogen). Slides were mounted with Mowiol 488 (Calbiochem), and DNA fibers were visualized using a Zeiss Axioplan confocal microscope. Images were analyzed using Zeiss LSM Image Browser software. Each data set is derived from measurement of at least 75 fibers.

### Protein analysis

To evaluate whether the point mutations used disrupted p21/CDK2 and p21/PCNA binding, we performed immunoprecipitations in the fraction (soluble) where p21/CDKs are usually found (17). Lysates were obtained using the flowing buffer: HEPES 25 mM, 100 mM NaCl, 10% glycerol, 2.5 mM NaF, 1 mM EDTA, Triton 0.5%, 0.5 mM sodium orthovanadate, 1 mM DTT 0.1 mM PMSF and protease inhibitors. After centrifugation, protein extracts were subjected to immunoprecipitations with two anti p21 antibodies (C19-Santa Cruz and AB1-EMD Millipore). To perform direct western blot analysis, samples were lysed in Laemmli buffer. Western blots were performed with anti-p21 (C19-Santa Cruz), anti-PCNA (PC10-Santa Cruz), anti-CDK2 (Santa Cruz), anti-actin (SIGMA) and KU70 (A9-Santa Cruz). Incubation with secondary antibodies (Sigma) and ECL detection (Amersham GE Healthcare) were performed according to manufacturer’s instructions.

### Statistical analysis

Frequency distribution of DNA fiber ratios was analyzed with GraphPad Prism 5 software. In non-Gaussian distributions, Mann–Whitney and Kruskal–Wallis tests where used for statistical analysis when comparing 2 and 3 variables, respectively. Statistical analysis of Y pols focal organization was performed in GraphPad inStat software using the Student’s *t*-test and one-way ANOVA test when applicable. Other calculations and graphics were performed using Microsoft Excel 2007.

## RESULTS

### UV triggers accumulation of replication stress markers when p21 degradation is prevented

**Figure 1.**
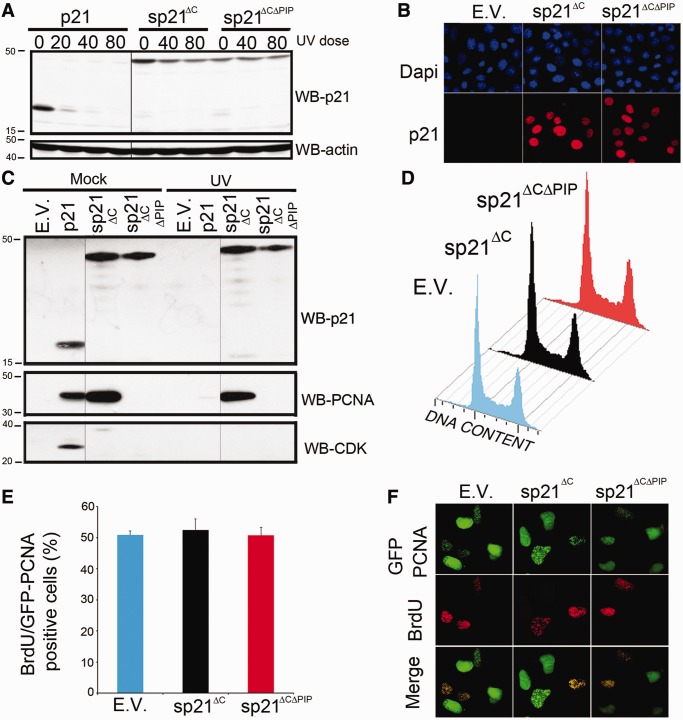
sp21^ΔC^ and sp21^ΔCΔPIP^ expression allows cell cycle progression. (**A**) sp21^ΔC^ and sp21^ΔCΔPIP^ are inefficiently degraded after UV irradiation. U2OS cells transfected with the indicated p21 constructs were irradiated, lysed 6 h after UV and used to determine p21 levels. (**B**) sp21^ΔC^ and sp21^ΔCΔPIP^ are nuclear. U2OS cells transfected with the indicated p21 constructs were fixed and immunostained for p21 (**C**) sp21^ΔC^ fails to interact with CDK, whereas sp21^ΔCΔPIP^ fails to interact with both CDK and PCNA. U2OS cells transfected with the indicated p21 constructs were UV irradiated (40 J/m^2^) and harvested 6 h later. Immunoprecipitations for p21 were performed as described in the ‘Materials and Methods’ section. The images shown for each blot correspond to the same gel and the same film exposure. (**D**) sp21^ΔC^ and sp21^ΔCΔPIP^ do not affect cell cycle distribution (FACS) in unstressed conditions. U2OS cell were cotransfected with f-GFP, and the indicated p21 constructs and the cell cycle profiles of the transfected populations were obtained by gating the f-GFP positive cells. (**E**) sp21^ΔC^ and sp21^ΔCΔPIP^ do not affect S phase progression. The percentages of U2OS transfected cells (GFP-PCNA positive cells) transiting S phase were determined by incorporating BrdU incorporation for 15 min before fixation. Similar results were obtained when using f-GFP as transfection marker (Supplementary Figure S7). (**F**) Representative fields of the experiment in (F) are shown. E.V. = Empty Vector.

p21 degradation after UV irradiation was reported by us and others [for detailed references see (1)]. To evaluate the biological relevance of p21 degradation, we used an N-terminal 6Myc-tagged p21 with blocked N-terminal ubiquitylation (13,18). As p21 stabilization triggers G1 arrest through the inhibition of Cyclin/CDKs complexes, we introduced point mutations to disrupt p21/CDK interaction (13). The resulting sp21^ΔC^ mutant showed increased stability after UV irradiation (Figure 1A), localized to the nucleus (Figure 1B) and did not bind CDK2 (Figure 1C), thus allowing cell cycle progression in unstressed conditions (Figure 1D–F). We also generated a sp21^ΔC^ mutant with a disrupted PCNA interacting protein (PIP) box by introducing point mutations as reported previously (13,19). As expected, sp21^ΔCΔPIP^ was stable (Figure 1A), nuclear (Figure 1B), failed to bind both CDK2 and PCNA (Figure 1C) and did not affect cell cycle progression in unstressed cells (Figure 1D–F).

**Figure 2.**
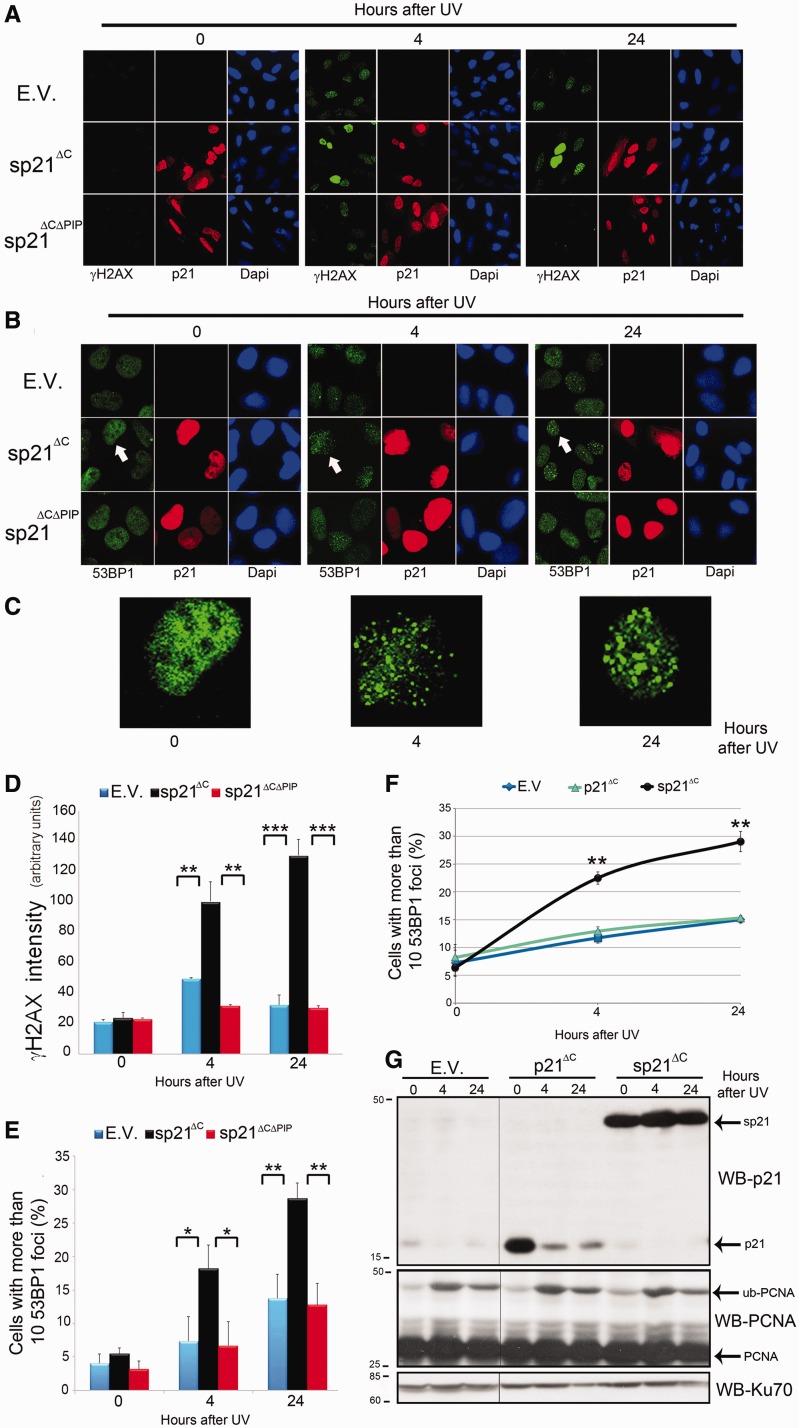
p21 degradation or the disruption of p21/PCNA interaction prevents increased accumulation of DNA-damage markers after UV irradiation. (**A**) U2OS cells transfected with the indicated p21 constructs were UV irradiated (5 J/m^2^), fixed at the indicated time points and subjected to γH2AX staining. (**B**) U2OS cells transfected with the indicated p21 constructs were UV irradiated (5 J/m^2^), fixed at the indicated time points and subjected to 53BP1 staining. (**C**) Zoomed images correspond to the cell indicated by an arrow in the p21 panels of Figure 2B. (**D**) Average γH2AX intensity was determined for 100 transfected nuclei for each time point in three independent experiments. (**E**) The percentage of cells with 10 or more consolidated 53BP1 foci was determined by analyzing 200 transfected cells in three independent experiments. (**F**) U2OS cells transfected with E.V., p21^ΔC^ and sp21^ΔC^ were UV irradiated (5 J/m^2^), fixed at the indicated time points and subjected to 53BP1 staining. In all, 200 nuclei in two independent experiments were analyzed. (**G**) Samples treated as in (F) were subjected to western blot with p21 and PCNA antibodies. KU70 was used as loading control. The images shown for each blot correspond to the same gel and the same film exposure. Significance of the differences between E.V. and each condition ***P < 0.001; **P < 0.01, *P < 0.05, no asterisk = NS—not significant, *P* > 0.05.

To explore the effect of forced p21 stabilization after UV irradiation, we evaluated the levels of a *bona fide* marker of replication stress, γH2AX (20). sp21^ΔC^, but not sp21^ΔCΔPIP^, expression caused a marked increase in the overall γH2AX intensity after UV irradiation (Figure 2A and D- notice increased pan nuclear intensity of γH2AX in representative panels for sp21^ΔC^). We also assessed the effect of sp21^ΔC^ on the accumulation of 53BP1 foci, which are generally associated with double strand breaks (DSBs) (21). sp21^ΔC^, but not sp21^ΔCΔPIP^, caused increased accumulation of cells with more than 10 53BP1 foci (Figure 2B, C and E). Notably, expression of degradable p21^ΔC^ did not augment 53BP1 foci when compared with control samples (Figure 2F and G), thus demonstrating that the observed phenotypes are triggered by p21 stabilization and are not a consequence of p21 overexpression. Isogenic HCT116 p21+/+ or p21−/− cells also showed similar percentages of cells with 53BP1 focal organization after 10 J/m^2^ of UV irradiation (percentages of 29.0 ± 3.4 and 31.0 ± 3.7, respectively—Supplementary Figure S1A). These results suggest that p21 degradation or the disruption of p21/PCNA interaction efficiently prevents accumulation of replication stress markers after UV irradiation.

### UV triggers increased S phase-associated genomic instability when p21 downregulation is prevented

**Figure 3.**
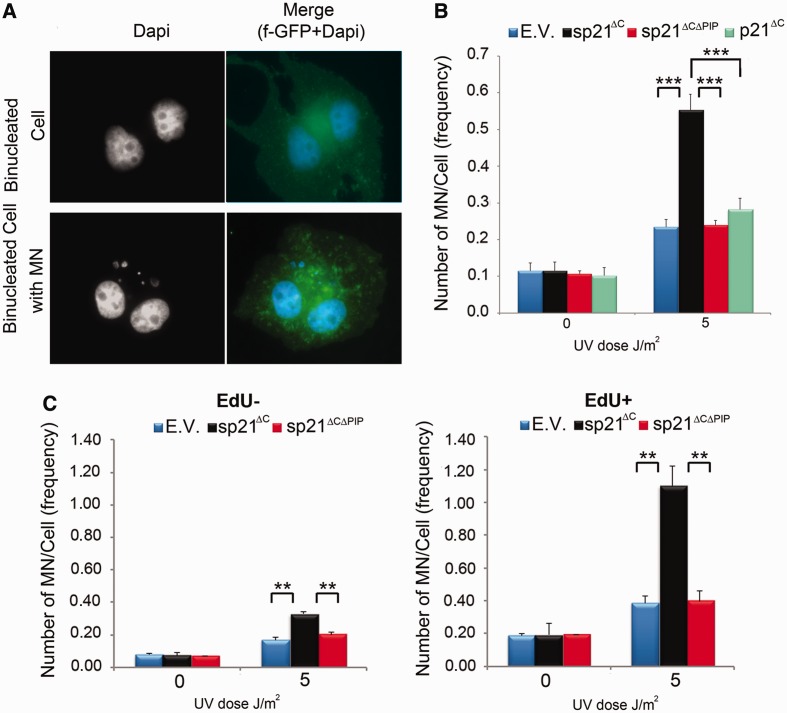
p21 degradation or the disruption of p21/PCNA interaction preserves genomic stability after UV irradiation. U2OS cells were transfected with GFP-PCNA or f-GFP, and the indicated p21 constructs were UV irradiated, trapped in cytochalasin B for 40 h, fixed and scored for micronuclei. (**A**) Representative binucleated cells with or without associated micronuclear structures are shown. (**B**) Average number of MN/cell was determined by scoring 300 f-GFP transfected nuclei in three independent experiments. (**C**) U2OS cells transfected with the indicated plasmids were UV irradiated and immediately pulsed labeled with EdU for 1 h. Samples were then processed as in (A). The number of MN/cell in EdU positive or negative cells was determined by analyzing 300 transfected binucleated cells in two independent experiments. Significance of the differences between E.V. and each condition ***P < 0.001; **P < 0.01, no asterisk = NS—not significant, *P* > 0.05.

As p21 stabilization causes UV-induced upregulation of both replication stress markers (Figure 2) and cell death [Supplementary Figure S1C–E and our previous report (9)], we evaluated the effect of sp21^ΔC^ on replication stress-associated genomic instability by quantifying MN formation (22,23). We observed a strong increase in MN formation after UV irradiation only when sp21^ΔC^ was expressed, but not with the sp21^ΔCΔPIP^ or degradable p21^ΔC^ constructs (Figure 3A and B). In agreement, HCT116 p21+/+ or p21−/− cells also showed similar levels of MN formation (percentages of cells with MN of 19.10 ± 0.02 and 20.90 ± 0.04, respectively—Supplementary Figure S1B). Interestingly, MN formation was much more frequent in sp21^ΔC^-expressing cells that were replicating DNA at the time of UV irradiation (EdU+ cells) than in EdU− cells (Figure 3C). These results demonstrate that replicating cells are highly sensitive to a failure to degrade p21 after UV irradiation, which triggers genomic instability as a consequence of sustained p21/PCNA interaction.

### The recruitment of specialized Y polymerases to replication factories is negatively regulated by p21

**Figure 4.**
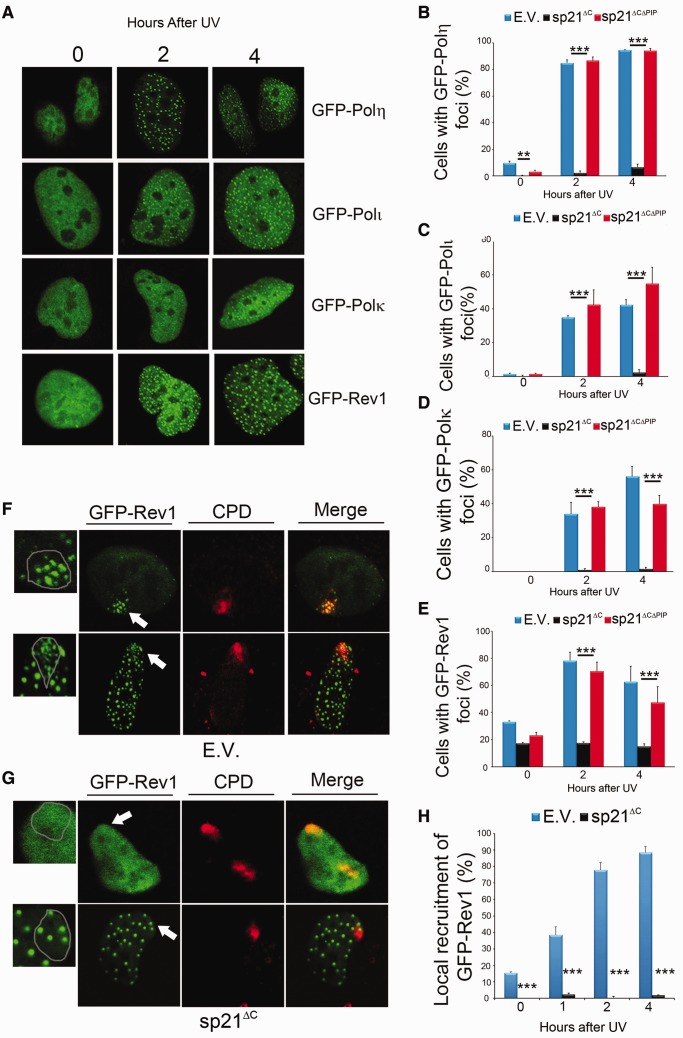
Persistent p21/PCNA interaction after UV irradiation prevents the organization of specialized Y polymerases into DNA replication-associated foci. (**A**) Representative nuclei of Hela cells showing that polymerases in the Y family are organized into focal structures on UV irradiation (40 J/m^2^). (**B**) Hela cells transfected with GFP-pol η and the indicated p21 construct were fixed at different time points after UV irradiation (40 J/m^2^), and the sub-nuclear distribution of pol η was determined in three independent experiments. Similar experiments were performed for pol ι (**C**), pol κ (**D**) and Rev1 (**E**) 200 cells/sample were counted, and cells with more than 10 foci per cell were scored as positive. (**F**) U2OS cells transfected with GFP-Rev1 and E.V. and were UV irradiated (120 J/m^2^) using polycarbonates filters. Antibodies specific against cyclobutane pirimidine dimmers were used to identify the damaged portion of nuclei. Representative images depict *de novo* UV-induced formation of GFP-Rev1 foci in samples with pan nuclear (upper panel) or focal (lower panel) organization of GFP-Rev1. (**G**) U2OS cells were transfected with GFP-Rev1 and sp21^ΔC^ and treated as in F. *De novo* focal organization of GFP-Rev1 was not observed after UV in cells with pan nuclear GFP-Rev1 (upper panels) or focal GFP-Rev1 (lower panels). In panels F and G, zoomed images depict GFP-Rev1 organization at sites of DNA damage corresponding to the areas indicated by arrows in the GFP-Rev1 panels. (**H**) Quantification of results shown in (F). In all, 100 nuclei were counted in two independent experiments. Significance of the differences between E.V. and each condition ***P < 0.001; no asterisk = NS—not significant, *P* > 0.05.

We have previously shown that p21 impairs PCNA ubiquitination and the formation of DNA replication-associated foci of pol η (9,13), and that PCNA ubiquitination is negatively regulated by stable p21 through its CDK binding domain (13). Accordingly, PCNA ubiquitination was not altered by the p21 mutants used in this study, which lack a functional CDK binding domain (Figure 2G). In contrast, the PCNA binding domain of p21 modulates pol η foci formation (9). Here, we extended our analysis to all specialized Y polymerases. This is particularly important because Y family pols are able to perform compensatory TLS when one member of the family is absent (24,25). Thus, a selective inhibitor of pol η activity would have completely different implications than a global inhibitor of TLS pols. We first confirmed that the focal organization of GFP-tagged pol η, pol ι, pol κ and Rev1 increases after UV irradiation (Figure 4A). sp21^ΔC^, but not sp21^ΔCΔPIP^, inhibited the focal organization of all Y polymerases (Figure 4B and D and Supplementary Figure S2), thus suggesting a global negative effect of p21 on the replication of damaged DNA. Intriguingly, we observed a differential effect of sp21^ΔC^ on Rev1 focal organization. Although Rev1 focal reorganization after UV irradiation was impaired by sp21^ΔC^ (Figure 4E), a constant 20% of cells scored positive for Rev1 foci throughout the whole time course of the experiment, despite sp21^ΔC^ expression. To get further insight into the nature of the GFP-Rev1 foci that resist sp21^ΔC^ expression, we UV-irradiated portions of nuclei using polycarbonate shields, which expose only discrete areas of the nucleus (26). In control samples, UV-triggered Rev1 foci formed within the UV irradiated area, regardless the state of Rev1 organization in the unirradiated nuclear background, which was pan nuclear (≅80% of the nuclei-representative image in Figure 4F, upper panel) or focal (≅20% of the nuclei-representative image in Figure 4F, lower panel). In strike contrast, UV–triggered Rev1 focal organization at damaged portions of the nucleus was always prevented by sp21^ΔC^ (Figure 4G, upper and lower panel). sp21^ΔC^ also prevented the interaction of PCNA with specialized Y polymerases in chromatinic immunoprecipitation assays (Supplementary Figure S3). Together, these results indicate that sp21^ΔC^ abolishes the UV-triggered recruitment of all Y family pols to replication factories.

**Figure 5.**
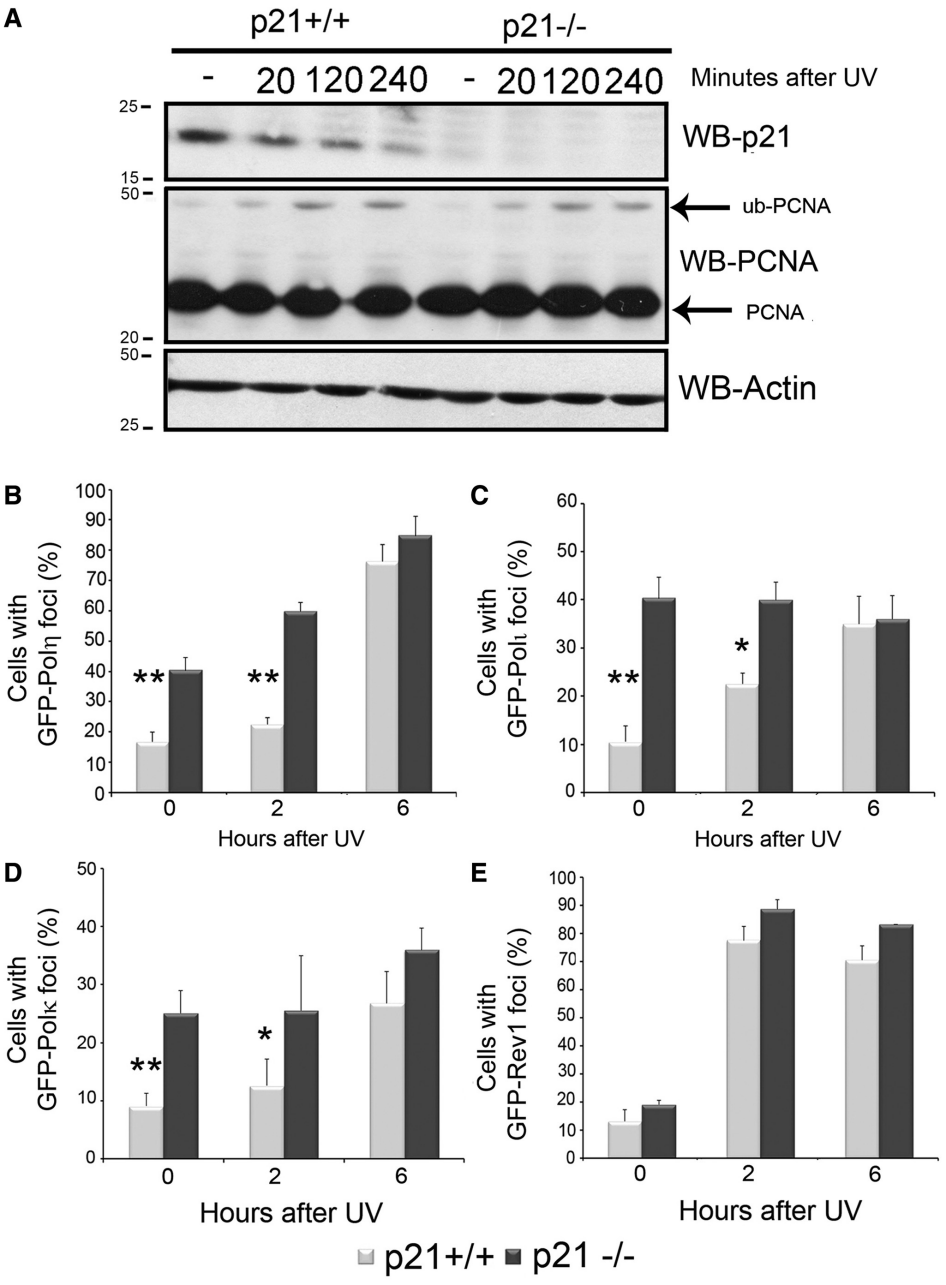
Endogenous p21 delays the recruitment of pols in the Y family to DNA replication-associated foci. (**A**) HCT116 p21+/+ and p21−/− were UV irradiated (30 J/m^2^) and lysed at the indicated time points. Western blots were performed using p21, PCNA and actin antibodies. (**B**) HCT116 p21+/+ and p21−/− cells transfected with GFP-pol η were fixed at different time points after UV irradiation (50 J/m^2^), and the sub-nuclear distribution of pol η was determined in three independent experiments. Similar experiments were performed for pol ι (**C**), pol κ (**D**) and Rev1 (**E**). In all, 200 cells/sample were counted. Significance of the differences between p21+/+ and p21−/− cells in each condition. **P < 0.01, *: *P* < 0.05, no asterisk = NS—not significant, *P* > 0.05.

When performing equivalent experiments with degradable p21, we observed that the expression of p21^ΔC^ slightly delayed—yet not repressed—pol η focal organization and did not impair pol η/PCNA interaction (Supplementary Figure S4). When using HCT116p21+/+ and p21−/− cells, we found that before and after stress, the focal organization of most Y pols (pol η, pol ι and pol κ) was upregulated in HCT116 p21 −/− cells (Figure 5B–E). This difference was evident only at early time points after UV irradiation and disappeared in a manner that correlated with p21 degradation (Figure 5A). Thus, UV-triggered degradation of p21 is required to achieve optimal organization of specialized Y polymerases at PCNA sites on DNA.

### The progression of DNA replication after UV irradiation is delayed by p21

**Figure 6.**
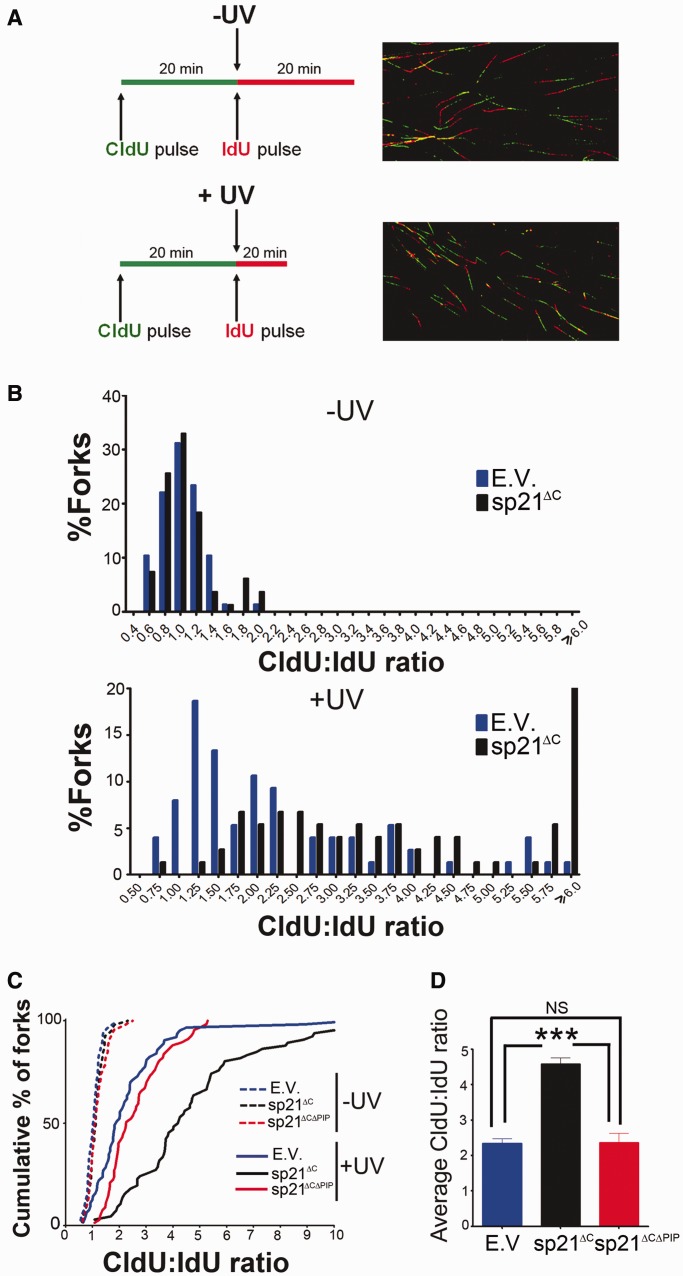
Persistent p21/PCNA interaction impairs the progression of ongoing replication forks after UV irradiation. (**A**) Schematic of the DNA fiber labeling experiment and representative images of DNA fibers. Unperturbed DNA replication results in ratios of CldU: IdU ∼1. Replication stalling during the second labeling period results in higher ratios. CldU, chlorodeoxyuridine; IdU, iododeoxyuridine. (**B**) U2OS cells were transfected with the indicated p21 constructs and UV irradiated (20 J/m^2^) when indicated. Samples were subjected to the DNA fiber labeling described in (A). Comparative distribution of ratios obtained in unirradiated controls (−UV) and UV-irradiated samples (+UV). Ratios higher than 6 were pooled together in the last category. (**C**) The data in (B) were plotted as a cumulative percentage of forks at each ratio. (**D**) Average ratios for the experiment shown in (B). Three independent experiments were analyzed obtaining similar results. Significance of the differences between E.V. and each condition ***P < 0.001.

Given that defects in TLS impairs the progression of replication forks after UV irradiation (27,28), we decided to analyze the effect of sp21^ΔC^ and sp21^ΔCΔPIP^ on the progression of replication forks immediately after UV irradiation. We used a DNA fiber spreading technique, a method that labels tracts of newly synthesized DNA *in vivo* (29). Two consecutive incorporations of different halogenated nucleotides, CldU and IdU, reveal two subsequent periods of DNA synthesis (in this study corresponding to before and after UV). The incorporation of these analogues into the DNA is visualized by fluorescence microscopy (Figure 6A, Supplementary Figures S5 and S6). A shorter second track indicates a delay or a block in the progression of DNA replication after UV irradiation. Average CldU/IdU ratios of ∼1 were obtained in sham-irradiated samples, and UV irradiation shifted the ratios to greater numbers in all samples (Figure 6B), as previously reported (16,27,28).

**Figure 7.**
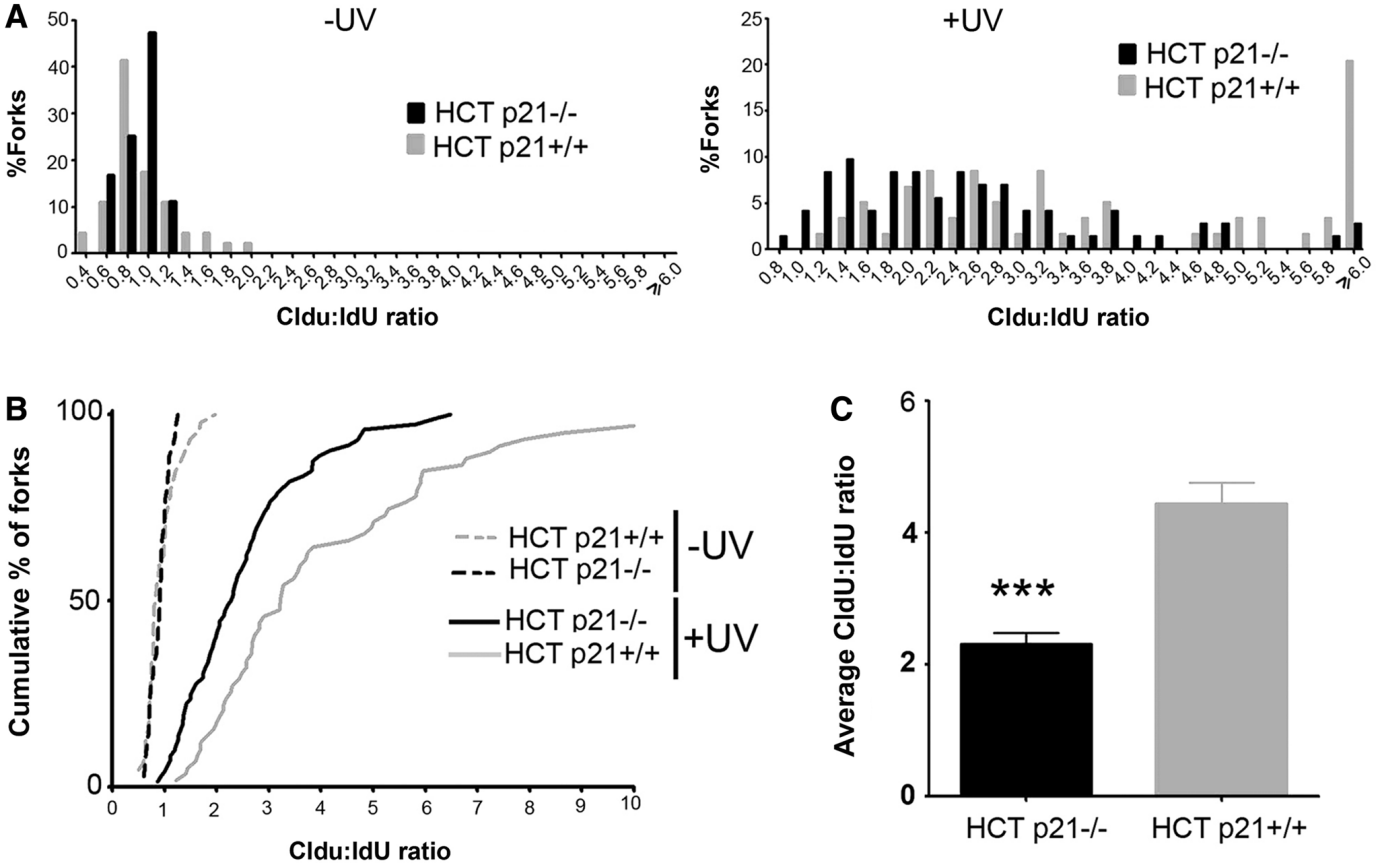
Endogenous p21 delays the progression of ongoing replication forks after UV irradiation. (**A**) HCT116 p21+/+ and p21−/− cells were UV irradiated (30 J/m^2^) when indicated. Samples were subjected to the DNA fiber labeling described in Figure 6. Comparative distribution of ratios obtained in unirradiated controls (−UV) and UV irradiated samples (+UV). Ratios higher than 6 were pooled together in the last category. (**B**) The data in (A) were plotted as a cumulative percentage of forks at each ratio. (**C**) Average ratios for the experiment shown in (A). Three independent experiments were analyzed obtaining similar results. Significance of the differences between p21+/+ and −/− cells; ***P < 0.001.

Although sp21^ΔC^ expression did not significantly alter unperturbed DNA replication (average CldU track length of 9.25 ± 2.75 for empty vector and 8.78 ± 2.70 for sp21^ΔC^), it impaired fork progression after UV irradiation (Figure 6B). To facilitate a direct comparison, the results were also plotted as cumulative percentages of forks at each ratio (Figure 6C). In this plot, the data corresponding to cells transfected with sp21^ΔCΔPIP^ were indistinguishable from control samples (Figure 6C). In agreement, average ratios were different only when sp21^ΔC^ was used (Figure 6D). We concluded that sp21^ΔC^, but not sp21^ΔCΔPIP^, impairs fork progression at early times after UV irradiation. Similarly, endogenous p21 also delayed the progression of replication forks after UV irradiation (Figure 7 and Supplementary Figure S6). Although this result suggested that, at early time points after UV irradiation, both degradable and stable p21 delays immediate replication events, it is important to highlight that these defects do not propagate equally to later replication events. When analysing cells expressing degradable p21 (either endogenous or exogenously overexpressed), no defects in S phase progression were observed 24 h after UV irradiation (Supplementary Figure S7A–C). However, sp21^ΔC^ caused persistent defects in S phase progression in a manner that was dependent on PCNA binding (Supplementary Figure S7A and B). Moreover, when trapping UV-irradiated cycling cells into a binucleated stage (that is reached only on finalization of DNA replication, mitosis and karyokynesis), we observed significantly fewer sp21ΔC-expressing cells getting to the binucleated stage (Supplementary Figure S7E). This suggests that sp21ΔC lead to permanent defects (at or before karyokinesis), which might result from irreversible deficiencies in DNA replication that occur when p21 is not removed from PCNA after UV irradiation. Thus, UV-triggered p21 degradation is required to promote damaged-DNA replication after UV irradiation and to preserve genomic stability.

## DISCUSSION

In this work, we provide evidence indicating that endogenous p21 delays early replication events after UV irradiation, and that its UV-triggered degradation is required to prevent latter defects in S phase progression. Conversely, forced p21 stabilization prevents the interaction of PCNA with Y polymerases complexes, which leads to persistent defects in S phase progression, accumulation of replication stress markers and genomic instability.

### The cellular response to UV irradiation requires p21 removal from PCNA at replication sites

Many years ago, Bunz and Volegstein showed that p53-dependent p21 upregulation prevents cell death after γ-irradiation by triggering cell cycle arrest (30). This scenario changes completely when UV light is used. Despite p53 accumulation, p21 levels do not rise after UV irradiation and are downregulated beyond basal levels in a p53-independent manner, particularly in cells that proliferate rapidly (1). The degradation of basal p21 is rather puzzling and suggests the existence of yet undescribed functions of p21 in cells (1). Our results suggest that the biological relevance of p21 degradation is associated with the facilitation of damaged DNA replication by means of PCNA/Y polymerases interaction. A failure to degrade p21 causes persistent alterations in S phase progression and increased 53BP1 focal accumulation and micronuclei generation, thus suggesting an increase in the number of DSBs when p21 is not degraded. As UV irradiation does not directly trigger DSBs formation, these DNA lesions would most certainly result from the collapse of replication forks under persistent replication stress. The fact that the pool of sp21^ΔC^ expressing cells that were transiting S phase just after UV irradiation is the one that preferentially accumulates micronuclei strongly supports this hypothesis.

Another intriguing implication of our findings is that endogenous p21 represses—or at least delays—initial TLS events after UV irradiation. We believe that this transient delay in TLS onset might be beneficial for cells. Using a plasmid-based assay, Livneh and colleagues (11) showed that p21 impairs the efficiency but increases the accuracy of TLS events. Although it is difficult to extrapolate a non-replicative model in which hosts cells are not subjected to UV irradiation (11) to our model, in which replication forks encounter DNA lesions caused by UV light, it is tempting to speculate on their combined implications. We propose that a balance between two opposite processes (PCNA-driven p21 degradation and impairment of specialized Y polymerases loading by p21 favors TLS accuracy, perhaps because a slow TLS onset might allow the selection of the proper specialized Y polymerase for each lesion.

p21 might regulate TLS through the control of PCNA ubiquitination (11,13). As PCNA ubiquitination is not regulated by the p21/CDK interaction (13), PCNA ubiquitination is not affected by p21^ΔC^ or sp21^ΔC^. Although more work is required to elucidate the complex relationship between p21 and PCNA ubiquitination, this article shows that p21 can directly control PCNA/Y polymerase interactions in a manner that is independent of PCNA ubiquitination.

### Competition and cooperation at replication forks that encounter damaged DNA

p21 degradation takes place in a dose-dependent manner (1,3,13,31), which is consistent with the likelihood of p21 degradation occurring at forks that encountered damaged DNA. Here, we show that forced maintenance of p21/PCNA interaction impairs the progression of ongoing replication forks and the recruitment of specialized Y polymerases to replication factories. This might be the consequence of the high affinity that the extended PCNA interacting region (PIR)—including PIP box and NLS (32)—of p21 has for PCNA (5,33). Many lines of evidence indicate that the p21 PIR efficiently and specifically displaces specialized Y polymerases from PCNA. First, sp21^ΔC^ specifically prevents the interaction of PCNA with pol η, pol ι and pol κ (this work), without affecting DNA polymerases involved in unperturbed DNA replication (8). Second, even endogenous levels of p21 impair the recruitment of overexpressed GFP-tagged Y polymerases to replication factories (this work). Third, the substitution of the pol κ -PIR for the one of p21 enhances the interaction of this chimera with PCNA (34). Fourth, the expression of p53 induced protein with death domain, a protein capable of disrupting the PCNA/p21 interaction at early times after UV irradiation, causes an increase in PCNA ubiquitination and PCNA interaction with pol η (35). Together, these data strengthen the link between p21 degradation and TLS onset. A timely removal of p21 from replication forks that encounters damaged DNA might be the key for accurate TLS.

### p21 fits the criteria to be defined as a negative regulator of TLS

The only currently accepted negative regulator of TLS is the deubiquitinase complex USP1/UAF1 (36). Our results highlight striking similarities in the regulation of USP1/UAF1 and p21, which strongly support the role of p21 in the negative modulation of TLS*.* First, both USP1 and p21 control the ubiquitylation of PCNA and the focal organization of specialized Y polymerases. USP1 directly removes the ubiquitin moiety from PCNA, negatively impacting on the focal organization of pol η (37). Instead, p21 negatively regulates TLS at least at two levels, controlling not only PCNA ubiquitylation but also the focal organization of specialized Y polymerases through the binding to CDKs (13) and PCNA (this work), respectively. These findings suggest that USP1 and p21 might cooperate to control or delay TLS events. Second, both p21 and USP1 are similarly regulated by UV irradiation and hydroxyurea (HU) treatment. Although UV irradiation prompts their fast destruction, the levels of both p21 and USP1 remain unchanged after hydroxyurea (HU) treatment (38,39). This might reflect different strategies used by cells in response to UV and HU, perhaps associated to the absence of direct induction of DNA damage by HU treatment (40). In any case, HU counteracts USP1 and p21 with alternative strategies: USP1 interaction with its activating partner UAF1 is eliminated by HU (37,38) and the interaction of p21 and PCNA is specifically prevented after HU (39). Third, the UV-triggered degradation of USP1 and p21 are not limited to S phase. It was recently shown that APC/CCdh1 triggers USP1 degradation specifically in G1 after UV irradiation (41). USP1 depletion might serve to pre-assemble TLS complexes and keep them ready for genomic replication (41). Interestingly, p21 is also degraded in G1 after UV irradiation, even in arrested cells (13). Although the function of specialized Y polymerases aggregates in G1 is yet unknown (42), it is clear that is actively favored by USP1 and p21 degradation (41,42). Collectively, negative regulators of TLS such as p21 and USP1 must be removed to promote damage bypass after UV irradiation. In the case of p21, this requirement is associated to the strong impact that PCNA/p21 interaction has on the cellular response to UV irradiation.

## SUPPLEMENTARY DATA

Supplementary Data are available at NAR Online: Supplementary Figures 1–7 and Supplementary Methods.

## FUNDING

National Institutes of Health (NIH) [R03 TW008924]; Agencia Nacional de Promoción Científica y Tecnológica (ANPCyT) (to V.G.); V.G. and G.S. are researchers from CONICET, and S.F.M., M.B.V. and M.H. received fellowships from CONICET and ANPCyT. Funding for open access charge: NIH [R03 TW008924].

*Conflict of interest statement.* None declared.

## Supplementary Material

Supplementary Data
