# Supplementary material for: UV-triggered p21 degradation facilitates damaged-DNA replication and preserves genomic stability
Source: Nucleic Acids Res. 2013 May 30;41(14):6942–51. doi: 10.1093/nar/gkt475 (PMC3737556; doi:10.1093/nar/gkt475)
Supplement: Supplementary Data [file supp_41_14_6942__index.html]

UV-triggered p21 degradation facilitates damaged-DNA replication and preserves genomic stability — UV-triggered p21 degradation facilitates damaged-DNA replication and preserves genomic stability — Supplementary Data 

# UV-triggered p21 degradation facilitates damaged-DNA replication and preserves genomic stability

## Supplementary Data

files

**Files in this Data Supplement:**

- Supplementary Data - pdf file
- Supplementary Data - doc file
- Supplementary Data - tif file
- Supplementary Data - tif file
- Supplementary Data - tif file
- Supplementary Data - tif file
- Supplementary Data - tif file
- Supplementary Data - tif file
- Supplementary Data - tif file
