# Supplementary material for: UV-triggered p21 degradation facilitates damaged-DNA replication and preserves genomic stability
Source: Nucleic Acids Res. 2013 May 30;41(14):6942–51. doi: 10.1093/nar/gkt475 (PMC3737556; doi:10.1093/nar/gkt475)
Supplement: Supplementary Data [file supp_gkt475_nar-00470-d-2013-File010.doc]

**Supplemental data**

**Supplemental Methods**

**Primers used to generate p21 mutations:** For the mutation of the CDK binding domain:

Forward: ctgcatccaggaggcccgtgagcgaaggaactccgcctttgtcaccga

Reverse: tcggtgacaaaggcggagttccttcgctcacgggcctcctggatgcag

That changed aa W49 to R, F51 to S and D52 to A. For the mutation of the PCNA binding domain:

Forward: cggcggcagaccagcgcgacagctgcctaccactccaaacgccggctg

Reverse: cagccggcgtttggagtggtaggcagctgtcgcgctggtctgccgccg

that change amino acids M147 to A, D149 to A and F150 to A. Both mutations were characterized previously .

**Chromatin immunoprecipitation of PCNA:** Chromatin immunoprecipitations were performed as previously described. U2OS cells were transfected and irradiated at 40 J/m2. Cells were rinsed twice with PBS and then extracted with 5 ml of CSK buffer (250 mM sucrose, 25 mM KCl, 10 mM HEPES [pH 8.0], 1 mM EGTA, 1 mM MgCl2) for 12 min. The CSK-extracted cells were fixed with 1% formaldehyde in PBS (4.5 ml) for 10 min. Then, 0.5 ml of 1 M glycine was added for 5 min to quench the cross-linking reaction. The cross-linked nuclei were rinsed with PBS and then lysed in 500 μl of IP lysis buffer (10 mM Tris-HCL [pH 7.5], 25 mM NaF, 20 mM NaCl, 1% NP-40, 1% Na-Deoxicholate. 0.1% SDS) freshly supplemented with protease and phosphatase inhibitors. Lysates were scraped from the plates and transferred into 1.5-ml Microfuge tubes. Samples were sonicated and clarified by centrifugation at 10,000 × g for 10 min. PCNA was immunoprecipitated overnight at 4°C with 5 μl of monoclonal PCNA antibody (PC-10 AC, Santa Cruz Biotechnology). Samples were washed, boiled to revert the crosslink and resolved in SDS PAGE for Western Blot analysis.

**Cytokinesis block proliferation index (CBPI):** Transfected cells were replated at low density and UV irradiated one day later. After 6 hs, cytochalasine B (6ug/ml) was added to the media. 24 hs later cells were fixed with PFA-2%sucrose 2% and, phalloidin and Dapi staining served to visualize the whole cell and nuclei respectively. 200 GFP positive binucleted cells were scored. The percentage of cell that had entered mitosis was calculated as the number of binucleated cells (arrested prior to cytokinesis by cytochalasin B) with respect to the total cells including both binucleated and mononucleated cells (cells that have not entered mitosis during the cytochalasin B treatment).

**Cell Viability and clonogenic assay:** To evaluate cell survival, U2OS cells were transfected, replated at low density and UV irradiated 1 day later. 72hs after irradiation transfected f-GFP positve cells were scored as viable or non-viable according to its nuclear structure and shape. 300 cells were counted in three independent experiments. For the clonogenic assay U2OS cells were transfected in 12-well plates, trypsinized 24hours later and replated onto 60-mm dishes in duplicates at a density of 250 cells per plate. The following day cells were UV irradiated at the indicated doses and incubated for 8-10 days. Colonies formed were fixed and stained with crystal violet and then counted in duplicates (colonies with more than 50 cells were scored as positive).

**γH2AX quantification:** To measure the average intensity of γH2AX in a given sample, photographs of random fields for γH2AX, p21 and DAPI were taken with a Zeiss Axioplan confocal microscope with the 60X water objective. All images within each experiment were acquired with the same exposure time and instrument settings and were processed equally without any further modification. Using the Image J software, nuclei were identified and delimited using the DAPI signal and transfected cells were selected using the p21 staining. The intensity of γH2AX in all transfected nuclei (either pan nuclear or focal) was calculated using the measure tool of the image J software.

**Supplementary Figures**

**Supplementary Figure 1**: Persistent p21/PCNA interaction after UV irradiation causes increased cell death after UV irradiation. A) HCT116 p21 +/+ and p21-/- cells were UV irradiated (10 J/m^2^) and 53BP1 foci accumulation was determined 4 hours later as described in Figure 2E. Representative panels of HCT 116 cells with and without 53BP1 focal organization are shown on the left. B) HCT116 p21 +/+ and p21-/- cells were UV irradiated (10 J/m^2^) and the micronuclei assay was performed as described in Figure 3. Representative panels of HCT 116 binucleated cells with and without micronuclei are shown on the left. C) Representative panels showing the shape and size of U2OS cells with intact nuclei and the contrasting image of one single fragmented nucleus. Bar: 15 μm B) U2OS cells were transfected with f-GFP and the indicated plasmids were UV irradiated with the indicated UV doses and fixed 72 hours later. 200 nuclei were scored in 3 independent experiments. C) U2OS were transfected with the indicated plasmid. 24 hours later samples were replated at colony forming density and UV irradiated (1.5 and 3 J/m^2^) 1 day later. Positive colonies were counted 8 days after UV irradiation. Samples were analyzed as duplicates and independent experiments were performed twice.

**Supplementary Figure 2:** Persistent p21/PCNA interaction after UV irradiation prevents the organization of specialized Y polymerases into DNA replication-associated foci. Experiments similar to the ones described in Figure 4 were performed in U2OS cells. A) Sub-nuclear distribution of pol η obtained from three independent experiments. Similar experiments were performed for pol ι (B), pol κ (C), and Rev1 (D) 200 cells/sample were counted. Significance of the differences between E.V. and each condition ***: p<0.001; **: p<0.01, *: p<0.05. Panels on the right of each plot are a representative nucleus (and zoomed fraction of the nucleus) showing the nuclear distribution for each polymerase in control and sp21 ^ΔC^ expressing cells after UV irradiation. When showing the nuclear distribution of Rev1 for sp21 ^ΔC^ expressing cells two representative images are shown: focal (21% of total cells) or pan nuclear (79%of total cells).

**Supplementary Figure 3**: Persistent p21/PCNA interaction impairs the formation of PCNA complexes with Y pols. A) U2OS cells were transfected with control or sp21^ΔC^ plasmids and GFP-pol η. 2 hours after UV irradiation (40 J/m2) samples were subjected to chromatin inmunoprecipitation using a monoclonal PCNA antibody as described in the Supplemental Material and Methods section. Similar experiments were performed with pol ι (B) and pol κ (C). The relative amounts of each polymerase in PCNA complexes was calculated by performing densitometric analysis of each band, normalization with respect to PCNA levels and is shown as folds relative to the unirradiated E.V. sample below each western blot.

**Supplementary Figure 4:** p21 degradation is required to achieve efficient polη focal organization and PCNA/polη interaction after UV irradiation. A) U2OS cells transfected with GFP-pol η and the indicated p21 construct were fixed at different time points after UV irradiation (40 J/m^2^) and the sub-nuclear distribution of pol η was determined in two independent experiments. Significance of the differences between cells expressing E.V. and p21 ^ΔC^  : **: p<0.01, *: p<0.05, no asterisk means no significant. The difference between E.V. and sp21 ^ΔC^  was always significant (***: p<0.001). B) U2OS cells were transfected with control or p21^ΔC^ and sp21^ΔC^ plasmids and GFP-pol η, 2 hours after UV irradiation (40 J/m2) samples were subjected to chromatin inmunoprecipitation using a monoclonal PCNA antibody as described in the Supplemental Material and Methods section.

**Supplementary Figure 5:** Persistent p21/PCNA interaction impairs the progression of ongoing replication forks after UV irradiation. U2OS cells were labeled with CldU and IdU following the protocol described in Figure 6A. Representative fields of labeled DNA fibers from U2OS cells transfected with the indicated p21 constructs are shown.

**Supplementary Figure 6:** Knockdown of endogenous p21 facilitates the progression of ongoing replication forks after UV irradiation. HCT116 p21+/+ and p21-/- cells were labeled with CldU and IdU following the protocol described in Figure 6A. Representative fields of labeled DNA fibers from HCT116 p21+/+ and p21-/- unirradiated and UV irradiated cells.

**Supplementary Figure 7:** Inefficient p21 degradation after UV irradiation impairs DNA replication and karyokinesis. A) U2OS cells co-transfected with the indicated p21 constructs and fGFP were or were not UV irradiated (5J/m2) and 24 hrs later EdU incorporation was performed for 15 minutes before fixation. Different fields were used to establish the number of EdU (+)/ transfected cells. B) Representative panels of UV irradiated fields are shown C) Experiments similar to the one described in A) were performed for HCT116 p21+/+ and -/- cells. D). U2OS cells transfected with the indicated p21 constructs were UV irradiated and the percentage of cells that reached Karyokinesis (and therefore finished S-phase and surpassed anaphase) was calculated using the CBPI. Representative mono and binucleated cells are shown. E) The number of binucleated cells/ transfected cells was determined scoring 200 transfected cells in three independent experiments. Significance of the differences between E.V. and each condition *: p<0.05, no asterisk= NS -not significant, p>0.05.
