# Supplementary figures and images for: UV-triggered p21 degradation facilitates damaged-DNA replication and preserves genomic stability
Source: Nucleic Acids Res. 2013 May 30;41(14):6942–51. doi: 10.1093/nar/gkt475 (PMC3737556; doi:10.1093/nar/gkt475)

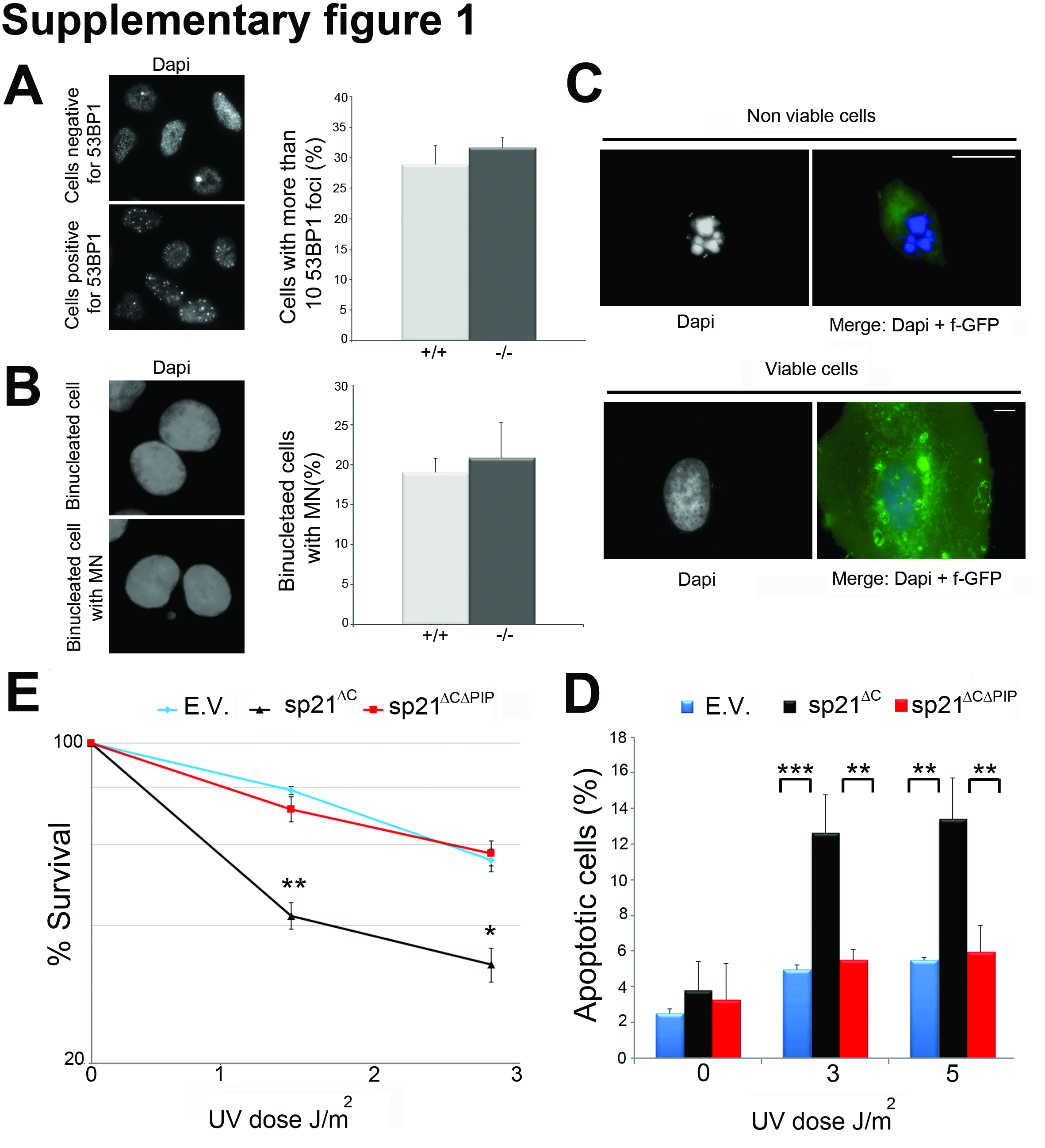

Supplement: Supplementary Data [file supp_gkt475_nar-00470-d-2013-File011.tif]

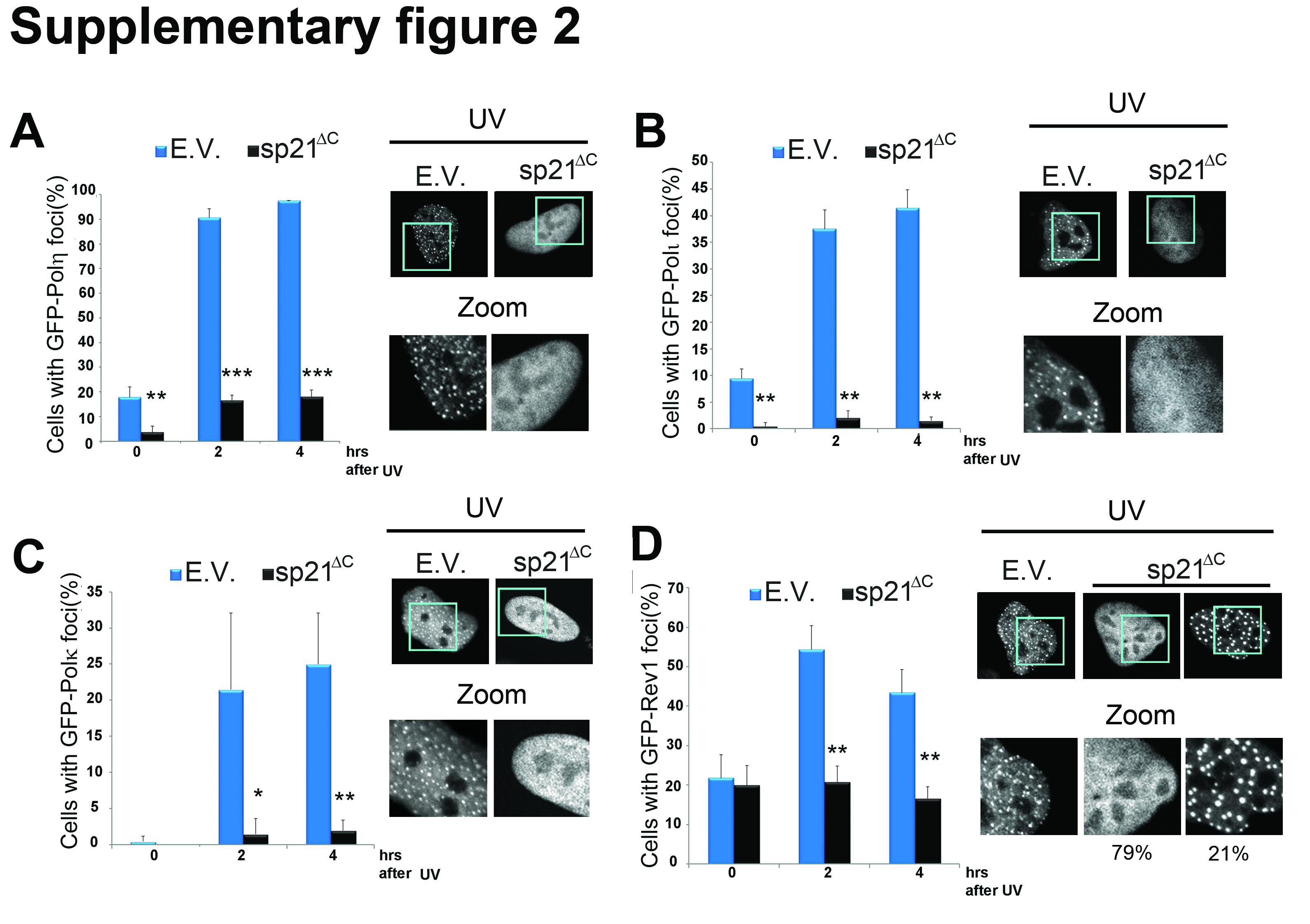

Supplement: Supplementary Data [file supp_gkt475_nar-00470-d-2013-File012.tif]

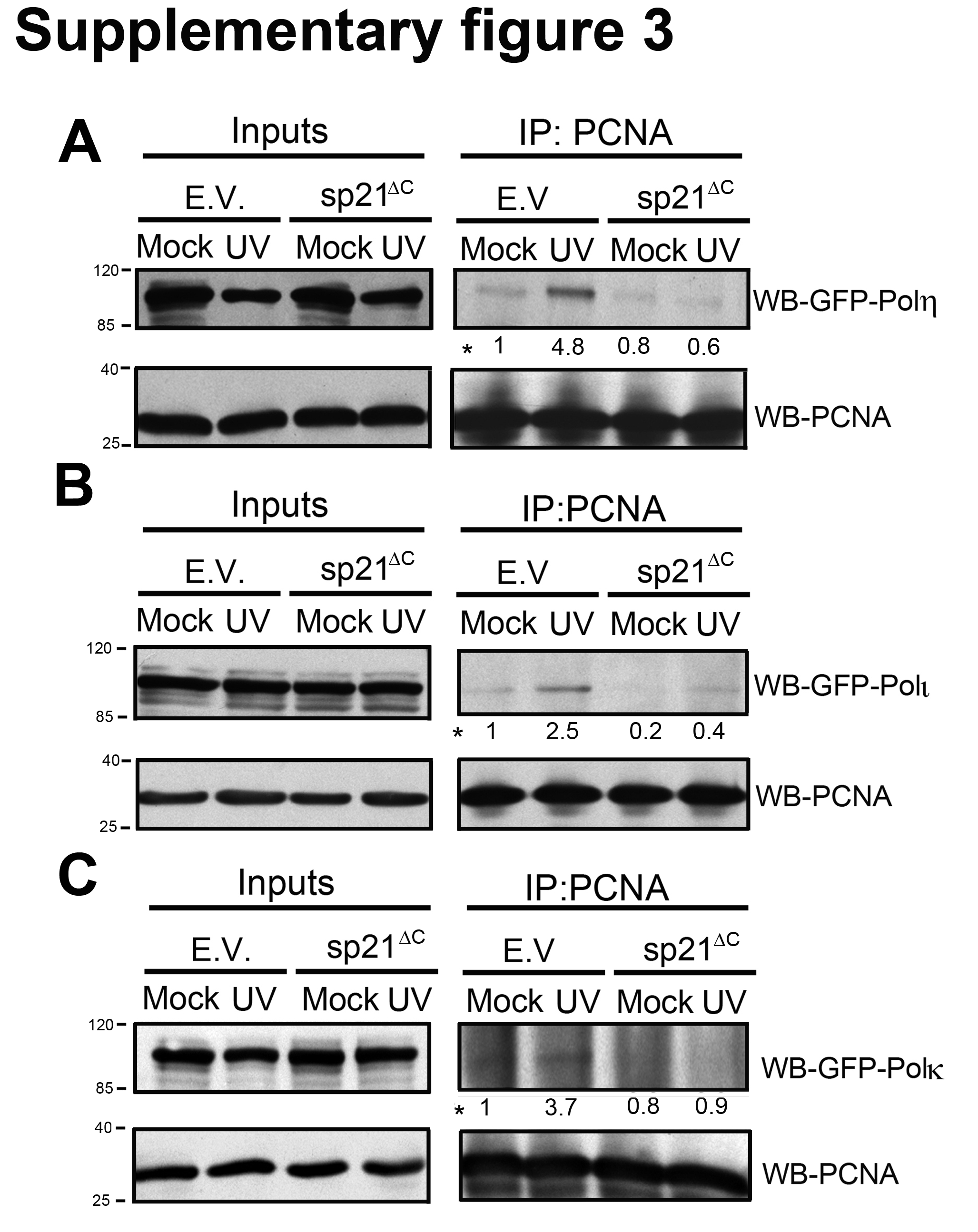

Supplement: Supplementary Data [file supp_gkt475_nar-00470-d-2013-File013.tif]

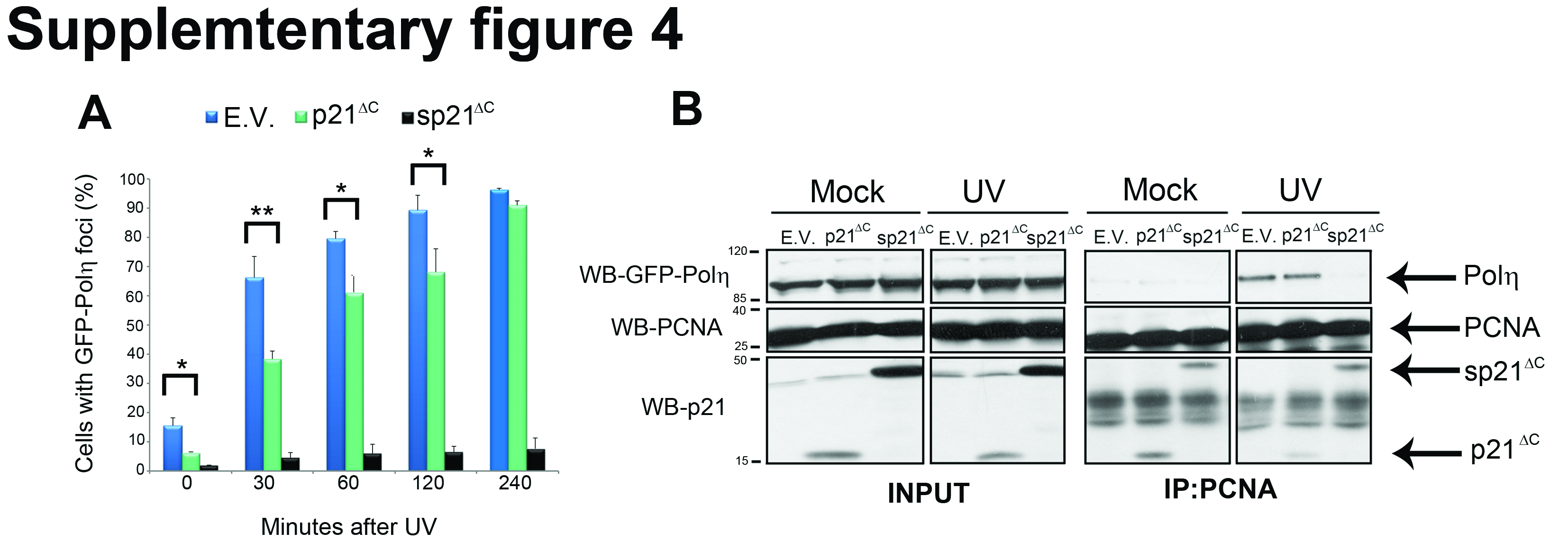

Supplement: Supplementary Data [file supp_gkt475_nar-00470-d-2013-File014.tif]

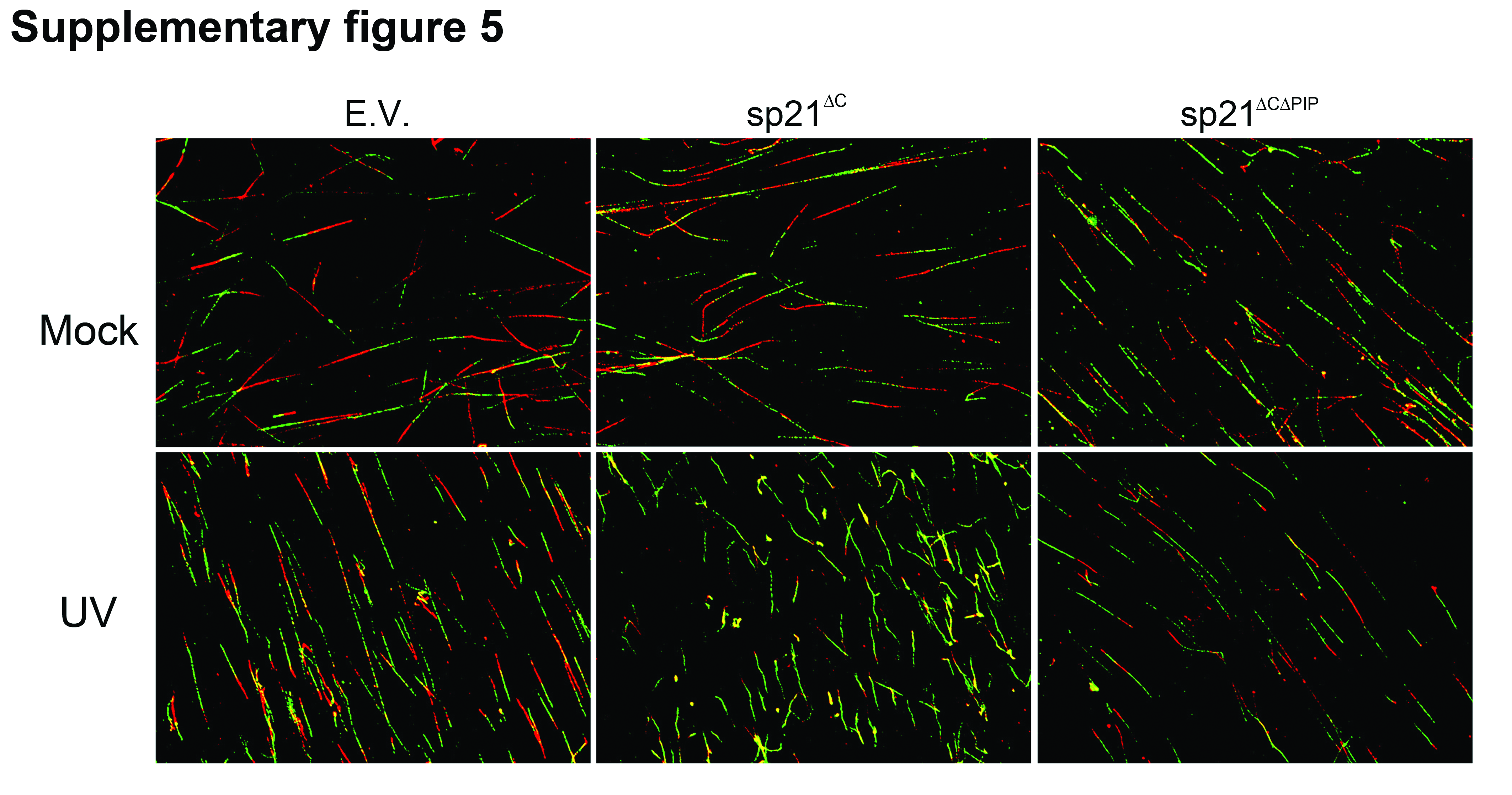

Supplement: Supplementary Data [file supp_gkt475_nar-00470-d-2013-File015.tif]

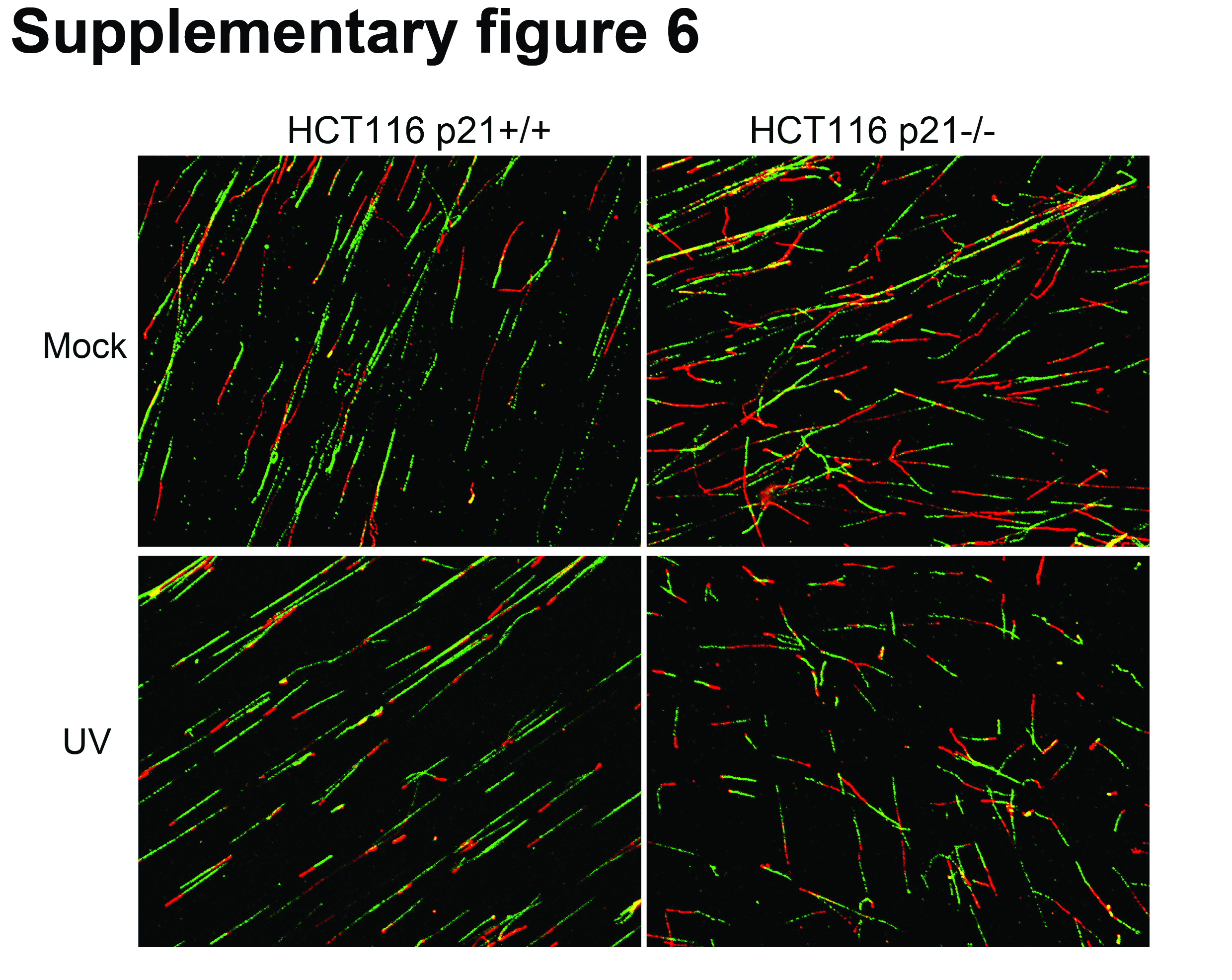

Supplement: Supplementary Data [file supp_gkt475_nar-00470-d-2013-File016.tif]

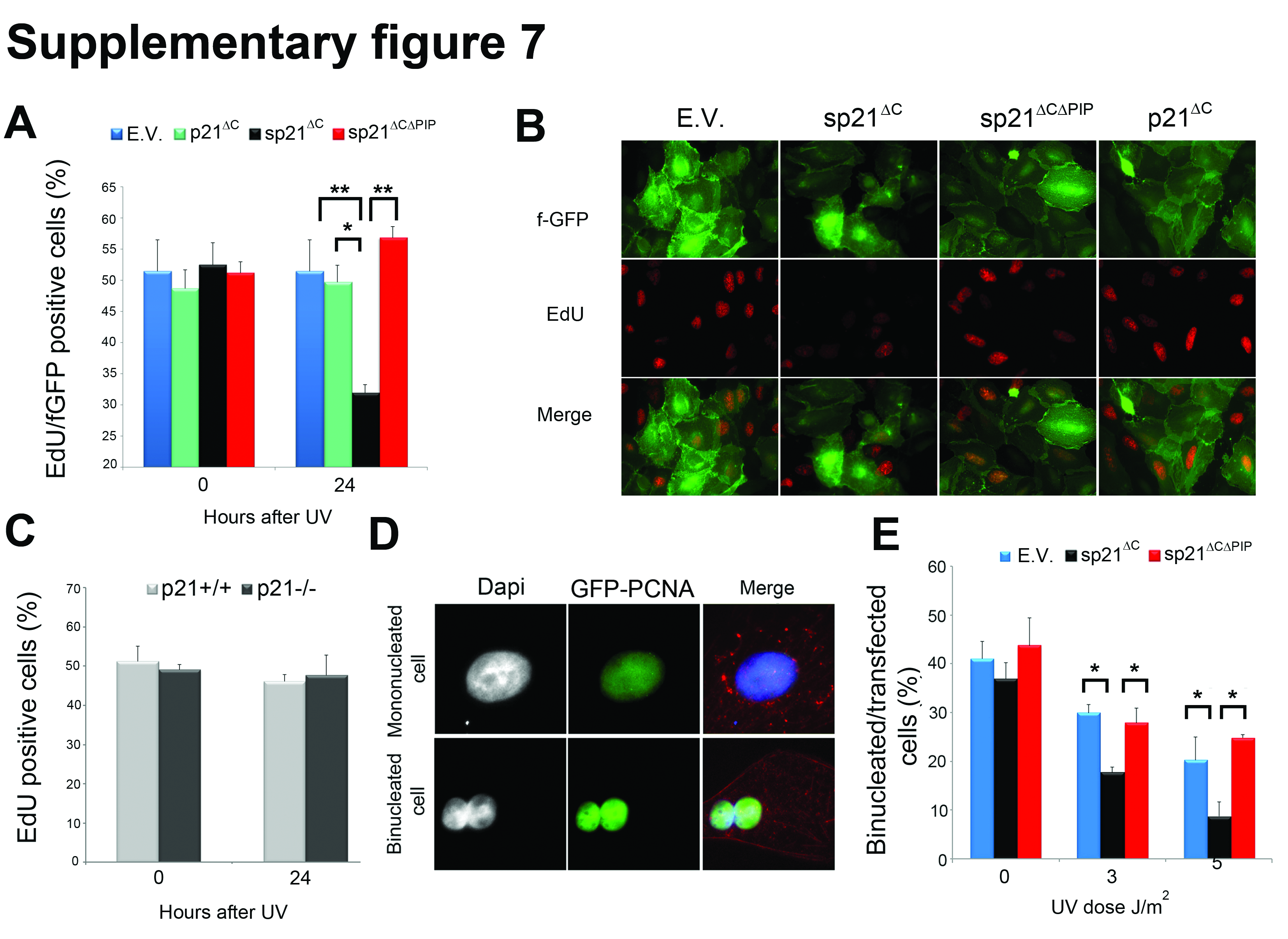

Supplement: Supplementary Data [file supp_gkt475_nar-00470-d-2013-File017.tif]
